# Involvement of Cholinergic and Opioid System in *γ*-Terpinene-Mediated Antinociception

**DOI:** 10.1155/2015/829414

**Published:** 2015-06-11

**Authors:** Flávia Franceli de Brito Passos, Everton Moraes Lopes, Jonas Moura de Araújo, Damião Pergentino de Sousa, Leiz Maria C. Veras, José Roberto S. A. Leite, Fernanda Regina de Castro Almeida

**Affiliations:** ^1^Medicinal Plants Research Center, Federal University of Piauí, Avenida Nossa Senhora de Fátima s/n, 64049-550 Teresina, PI, Brazil; ^2^Department of Pharmaceutical Sciences, Federal University of Paraíba, University City, s/n, 58051-900 João Pessoa, PB, Brazil; ^3^Center for Research on Biodiversity and Biotechnology, Federal University of Piauí, Avenida São Sebastião, No. 2819, 64202-020 Parnaíba, PI, Brazil

## Abstract

The literature shows that the monoterpenes are great candidates for the development of new drugs for the treatment of various pathological processes, including painful conditions. The gamma terpinene (*γ*-TPN) is a monoterpene present in plant species that have multiple pharmacological properties and has structural similarity to antinociceptive monoterpenes, such as limonene and alpha-phellandrene. The *γ*-TPN molecular mass was evaluated by mass spectrometry and showed a pseudomolecular ion with *m*/*z* 137.0 Da. The animals did not present any signs of acute toxicity at 2 g/kg, p.o. *γ*-TPN (1.562 to 50 mg/kg, p.o.) showed an antinociceptive effect in the formalin, capsaicin, and glutamate tests. *γ*-TPN has antinociceptive action when administered by others routes in glutamate test. To eliminate a possible sedative effect of *γ*-TPN, the open field and rota-rod test were conducted and the *γ*-TPN did not show muscle relaxant activity or central depressant effect. To investigate the mechanisms of action, the animals were pretreated with naloxone, glibenclamide, atropine, mecamylamine, or L-arginine in the glutamate test. *γ*-TPN antinociception was inhibited in the presence of naloxone, glibenclamide, atropine, and mecamylamine. The results suggest that the *γ*-TPN (p.o.) produced antinociceptive effect in models of chemical nociception through the cholinergic and opioid systems involvement.

## 1. Introduction

The essential oils are volatile, natural, complex, and characterized by the presence of a strong odour and are formed by aromatic plants as secondary metabolites. Their pharmacological properties include virucidal, fungicidal, analgesic, anti-inflammatory, and antispasmodic [1, 2]. In turn, the monoterpenes represent a group of naturally occurring organic compounds named “terpenes,” whose basic structure consists of two linked isoprene units, which are formed by 5-carbon-base (C5) each. Moreover, monoterpenes are the most representative molecules constituting about 90% of essential oils content and have a great variety of structures [1].

For more than two decades, many researchers have studied the analgesic potential of monoterpenes through of the in vivo and in vitro assays [3]. Studies of proposed analgesic-like activity mechanisms have been conducted with some monoterpenes, acting on several receptors, including opioids, adenosine A1 and A2, or cholinergic M2, producing changes in K+ channels, inhibition of peripheral mediators, and nitric oxide synthesis modulation, among others mechanisms [4–11]. The great diversity of mechanisms that may be associated with the analgesic effect of these monoterpenes is amazing, and several monoterpenes presented more than one mechanism of action that can be related to this effect [3].

**Figure 1:**
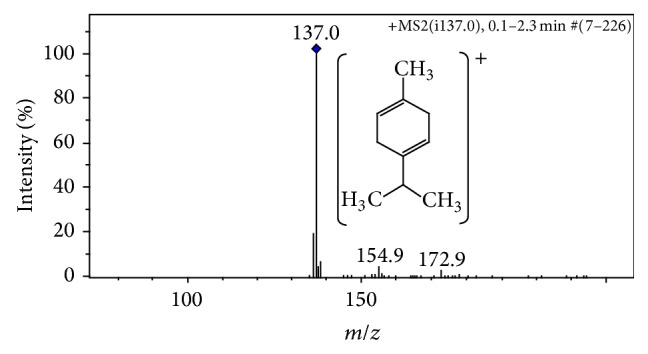
ESI+ MS/MS spectra of *γ*-terpinene (1-methyl-4-isopropyl cyclohexadiene-1,4) confirm the *m*/*z* ratio characteristic, [M + H]^+^ = 137.01 Da.

The *γ*-terpinene (*γ*-TPN) (1-methyl-4-isopropyl cyclohexadiene-1,4) (Figure 1) is a monoterpene present in several plant species pharmacologically active, for example, in the essential oils from* Protium icicariba* (DC.) Marchand [12],* Citrus deliciosa* Tenore [13], and* Origanum onites* L. [14], among others. The presence of unsaturation in its cyclic chain structure confers the olefin characteristic for the *γ*-TPN, which allows easier absorption through biological membranes, due to the lipossolubility [15]. In acute toxicity tests, *γ*-TPN showed LD_50_ in rats of 3.65 g/kg and the acute dermal LD_50_ in rabbits exceeded 5 g/kg [16].

Then, considering the structural similarity with other monoterpenes with antinociceptive activity, such as limonene [17] and *α*-phellandrene [18], as well as no evidences of antinociceptive activity of *γ*-TPN have been reported, the aim of the present work was to investigate the antinociceptive potential of *γ*-TPN in experimental nociception models in mice, as well as the possible contribution of cholinergic (i.e., muscarinic and nicotinic acetylcholine receptors) and opioidergic (i.e., opioid receptors) systems, K^+^_ATP_ channels, and the L-arginine-nitric oxide pathway to the pharmacological activity.

## 2. Materials and Methods

### 2.1. The Monoterpene *γ*-TPN

The molecular mass was confirmed by mass spectrometry in positive electrospray ionization mode (AmaZon SL, Bruker Daltonics, Bremen, Germany), under the following conditions: capillary voltage of 2,000 V; temperature of 250°C; 12 psi of pressure to nebulizer; and 10 L/min of flux to dry gas. The mass spectra were acquired in mass range of *m*/*z* at 70–500 Da. The 137 Da ions were selected within an isolation width of 2 u and scans were accumulated with variable RF signal amplitudes. The *m*/*z* scale of the mass spectrum was calibrated using the external calibration standard G2421A electrospray “tuning mix” (Agilent Technologies, Santa Rosa, USA).

### 2.2. Animals

In the acute toxicity evaluation, female Wistar rats (180–200 g, *n* = 6 per group) were used. The acute pain tests were carried out on male Swiss mice (20–30 g, *n* = 6–9), reared at the Medicinal Plants Research Center of the Federal University of Piauí. The animals were housed at 22 ± 1°C under a 12 h light-dark cycle with free access to food and water. Animals were acclimatized at least 1 h before testing and were used only once throughout the experiments. The protocols were approved by the Institutional Ethics Committee (Ethics Committee on Animal Experimentation/UFPI, n°. 008/12) and were carried out in accordance with the current guidelines for the care of laboratory animals and the ethical guidelines for investigation of experimental pain in conscious animals [19].

### 2.3. Drugs and Chemicals

The following substances were used: *γ*-TPN, glutamic acid, glibenclamide, mecamylamine, nicotine, atropine, pilocarpine, MK 801, capsaicin, N*ω*-nitro-L-arginine (L-NOARG), L-arginine (all purchased from Sigma-Aldrich, USA), morphine, naloxone hydrochloride, and diazepam (purchased from Cristália, SP, Brazil). Doses of antagonists were based on previous studies from our group. For the pharmacological studies, the *γ*-TPN was suspended in 2% Tween 80 in 0.9% NaCl (10 mL/kg). The doses were reported as milligrams of *γ*-TPN per body weight (mg/kg). The doses range was 1.56 to 50 mg/kg. The results of lower or upper doses for some protocols were not shown, due to similarity in pharmacological effects between the previous and next doses. Capsaicin was prepared in 2% Tween 80 solution in 2% ethanol. The *γ*-TPN was administered orally (p.o.), intrathecally (i.t.), intracerebroventricularly (i.c.v.), and intraplantarly (i.pl.) at different doses in order to construct the dose-response curves.

### 2.4. Determination of Acute Toxicity in Rats

The toxicological evaluation of *γ*-TPN was performed by the fixed dose procedure, recommended by the Organization for Economic Cooperation and Development (OECD) number 420 [20]. The animals were divided into 2 groups of 5 animals each; one test group was orally treated with *γ*-TPN at a dose of 2 g/kg, and a control group orally treated with 2% Tween 80 in 0.9% NaCl solution. Immediately after administration, the animals were evaluated clinically and behaviorally with greater attention during the first 4 hours after administration, as recommended by the protocol of recognition and evaluation of clinical signs of OECD [21]. Then, the evaluations were performed daily, and the weights of the animals were obtained during 14 days. After toxicological investigation, the animals were euthanized, the relative weights of internal organs were determined, and the serum biochemical and hematological parameters were performed, as well as the quantification of reduced glutathione and catalase activity.

### 2.5. Antinociceptive Effect of *γ*-TPN

#### 2.5.1. Effect of *γ*-TPN in Formalin Test

Mice were orally treated with *γ*-TPN (6.25, 12.5 and 25 mg/kg) or vehicle 1 h before the test. Morphine (5 mg/kg) was subcutaneously administered 30 min before the test as a positive control. The right hind paw was injected with formalin (20 *μ*L, 2%) in the intraplantar region. Nociception was evaluated by quantifying paw licking time during the first 5 min (first phase) and at 15–30 min (second phase) [22].

#### 2.5.2. Effect of *γ*-TPN in Capsaicin Test

Mice were treated with *γ*-TPN (12.5, 25 and 50 mg/kg, p.o.), vehicle, or morphine (5 mg/kg, s.c.). One hour (p.o.) and 30 min (s.c.) after these treatments, the right hind paw was injected with capsaicin (2 *μ*g/20 *μ*L/paw). Nociception was assessed immediately after injection and quantified by paw licking time during a 5-min period [23].

#### 2.5.3. Effect of *γ*-TPN in Glutamate Test

The procedure used was similar to a previously described method [24]. Mice received an injection of glutamate by intraplantar route (20 *μ*mol/paw) after a previous treatment with *γ*-TPN by oral (1.56, 3.125, or 6.25 mg/kg, 60 min beforehand), intrathecal (i.t., 5–20 *μ*g/site, 15 min beforehand), intracerebroventricular (i.c.v., 5–20 *μ*g/site, 15 min beforehand), or intraplantar (10 and 20 *μ*g/paw, coadministered with glutamate) routes. Control animals were treated with vehicle by oral, spinal (i.t., 5 *μ*L/site), supraspinal (i.c.v., 1 *μ*L/site), or intraplantar (20 *μ*g/paw, coadministered with glutamate) routes before glutamate injection. MK801 (0.03 mg/kg, i.p.) was used as positive control and administered 30 min before glutamate.

### 2.6. Investigation of Mechanisms of *γ*-TPN-Induced Antinociceptive Action

In order to elucidate the mechanisms underlying *γ*-TPN-induced antinociception, mice were pretreated intraperitoneally in the glutamate model (*n* = 6–9) with naloxone (2 mg/kg), a nonselective antagonist of opioid receptor; glibenclamide (3 mg/kg), an antagonist of K^+^_ATP_ channels; atropine (1 mg/kg), an antagonist of muscarinic receptors; mecamylamine (2 mg/kg), an antagonist of nicotinic receptors; and L-arginine (600 mg/kg), a substrate for NO biosynthesis. The doses of these drugs were selected in according to literature data and previous results from our laboratory [25–27].

### 2.7. Measurement of Motor Performance

#### 2.7.1. Open Field Test

The apparatus for the open field test consists of an acrylic box with transparent walls (30 cm × 30 cm × 15 cm) and a black floor divided into nine squares of equal area. One day before the experiment, mice were placed in the box for adaptation. The mice were treated with *γ*-TPN (12.5 and 25 mg/kg, p.o.), vehicle (p.o.), or diazepam (4 mg/kg, i.p.) 30 min and 1 h before individual observation, and the number of crossings (crossed squares with all paws) was counted during a 5 min session [28].

#### 2.7.2. Rota Rod Test

The rota-rod (Model RR-2002, Insight equipment) consisted of a 2.5 cm diameter bar, subdivided into four compartments by 25 cm diameter disks, rotating at 14 revolutions per minute. Mice were submitted to a trial 24 h before experiment, in order to eliminate those animals that did not remain on the bar for three consecutive periods of 60 s. The mice were treated with *γ*-TPN (12.5 and 25 mg/kg, p.o.), vehicle (p.o.), or diazepam (4 mg/kg, i.p.) 0.5 and 1 h before the experiment. Results are expressed as the time (s) that mice remained on the rota-rod, and the cut-off time used was 60 s [29].

### 2.8. Statistical Analysis

The results were expressed as the mean ± SEM and analyzed by one-way analysis of variance, followed by Tukey's or Bonferroni's post hoc tests. Significative differences among groups were considered when *P* < 0.05 (GraphPad Prism version 5.00 for Windows, GraphPad Software, San Diego, CA, USA, http://www.graphpad.com/).

## 3. Results

### 3.1. Mass Spectrometry

The MS analysis of *γ*-TPN revealed a pseudomolecular ion signal with *m*/*z* 137.0 Da [M + H]^+^ (Figure 1), in acordance to its molecular weight of 136.234 Da, confirming the identity and purity of *γ*-TPN.

### 3.2. Evaluation of Acute Toxicity in Rats

The *γ*-TPN at 2 g/kg (p.o.) did not demonstrate any sign of evident toxicity during the 14 days of observation as well as any death was observed. Therefore, the LD_50_ of *γ*-TPN was not possible to be determined. Furthermore, no behavioral and clinical alterations after administration of *γ*-TPN were observed. The *γ*-TPN did not alter the internal organs relative weights, the biochemical parameters, and the catalase and reduced glutathione, when compared to vehicle (data not shown).

### 3.3. Antinociceptive Effect of *γ*-TPN

#### 3.3.1. Effect of *γ*-TPN in Formalin Test

As shown in Table 1, *γ*-TPN at 12.5 and 25 mg/kg (p.o.) significantly reduced the licking time of the stimulated paw in both phases of the test when compared with vehicle (^**^ *P* < 0.01; ^***^ *P* < 0.001), while the dose of 6.25 mg/kg (p.o.) did not do this.

#### 3.3.2. Effect of *γ*-TPN in Capsaicin Test

**Figure 2:**
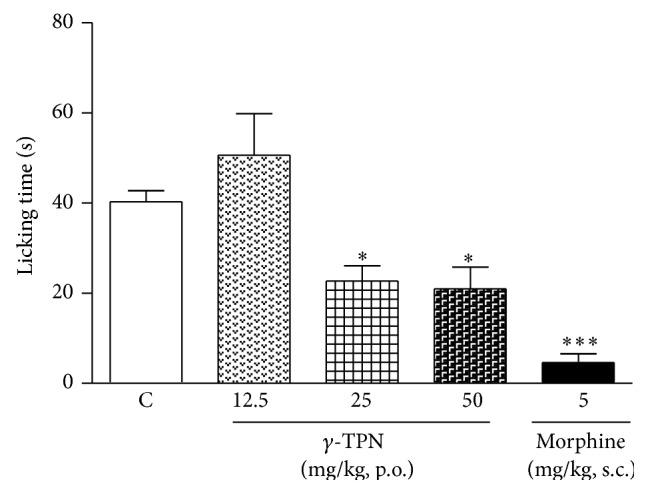
Effect of the *γ*-TPN on capsaicin-induced nociception in mice. Animals were treated with *γ*-TPN 60 min (p.o.) before capsaicin test. Data represent the mean ± SEM of 6–9 mice. ^***^ *P* < 0.001, ^*^ *P* < 0.05 compared with vehicle.

The effect of *γ*-TPN against capsaicin-induced nociception in mice is shown in Figure 2. A significant reduction in time length spent on licking the paw was observed in mice administered with *γ*-TPN (25 and 50 mg/kg p.o.) compared with vehicle (^*^ *P* < 0.05), indicating an antinociceptive effect in neurogenic pain. Morphine (5 mg/kg s.c.) was used as positive control and showed a decrease of the response when compared with vehicle (^***^ *P* < 0.001).

#### 3.3.3. Effect of *γ*-TPN in Glutamate Test

**Figure 3:**
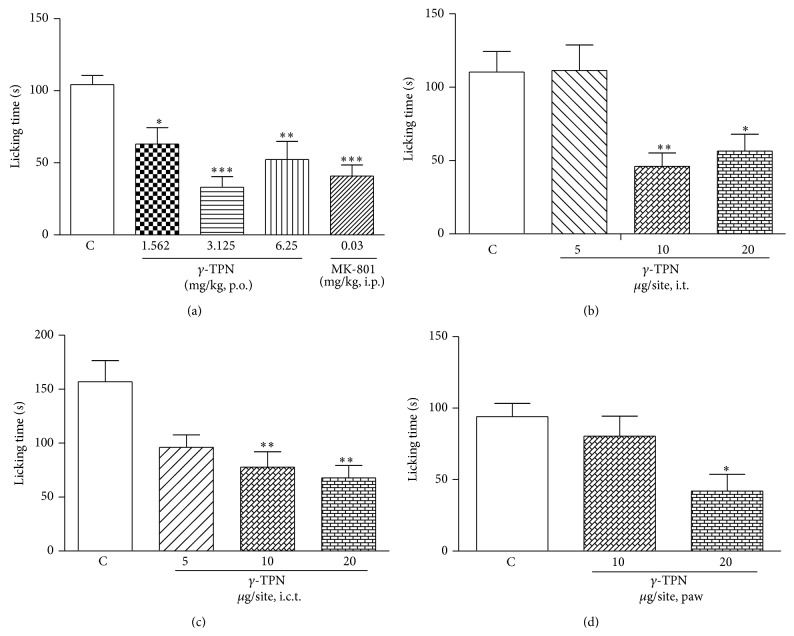
Effect of the *γ*-TPN administered orally (a), intrathecally (b), intracerebroventricularly (c), or intraplantarly (d) against licking induced by intraplantar injection of glutamate (20 *μ*mol/paw) in mice. Each column represents the mean of 6–8 animals and the error bars indicate the S.E.M. Control values (c) indicate the animals injected with saline and the asterisks denote the significance levels ^*^ *P* < 0.05, ^**^ *P* < 0.01, and ^***^ *P* < 0.001 when compared with control group values (one-way ANOVA followed by Tukey's test).

The results in Figures 3(a), 3(b), 3(c), and 3(d) show that *γ*-TPN given either systemically (p.o.) (1.56, 3.125, and 6.25 mg/kg) (^*^ *P* < 0.05, ^**^ *P* < 0.01, and ^***^ *P* < 0.001) or centrally (i.t or i.c.v) (10 and 20 *μ*g/site) (^**^ *P* < 0.01, ^*^ *P* < 0.05) caused significant inhibition of glutamate-induced nociception when compared to vehicle, while intraplantar treatment with *γ*-TPN (20 *μ*g/site) partially inhibited glutamate-induced nociception (^*^ *P* < 0.05).

### 3.4. Analysis of Possible Antinociceptive Mechanisms of *γ*-TPN

**Figure 4:**
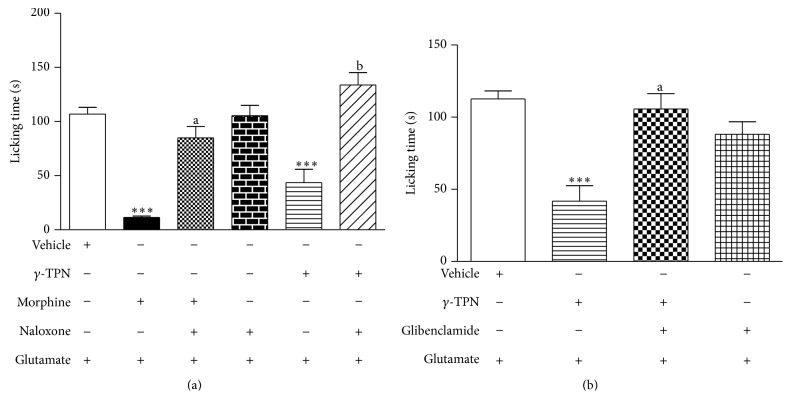
Effect of the *γ*-TPN (3.125 mg/kg, p.o.) against the action of naloxone (2 mg/kg, i.p.) (a) and glibenclamide (3 mg/kg, i.p.) (b) on glutamate-induced nociception (20 *μ*L, 20 *μ*mol/paw) in mice. Data represent mean ± SEM of 6–9 mice. Figure 4(a), the symbols indicate the level of significance: ^**^ *P* < 0.001 compared with vehicle, ^a^ *P* < 0.001 compared with the morphine group, ^b^ *P* < 0.001 compared with *γ*-TPN group. Figure 4(b), ^***^ *P* < 0.001 compared with vehicle, ^a^ *P* < 0.01 compared with *γ*-TPN group; + present treatment; − missing treatment (one-way analysis of variance, Bonferroni's test).

**Figure 5:**
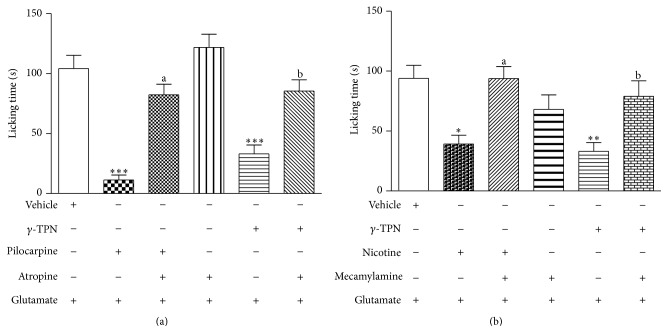
Effect of the *γ*-TPN (3.125 mg/kg, p.o.) against the action of atropine (1 mg/kg, i.p.) (a) and mecamylamine (2 mg/kg, i.p.) (b) on glutamate-induced nociception (20 *μ*L, 20 *μ*mol/paw) in mice. Data represent mean ± SEM of 6–9 mice. In Figure 5(a), the symbols indicate the level of significance: ^***^ *P* < 0.001 compared with vehicle, ^a^ *P* < 0.001 compared with the pilocarpine group, and ^b^ *P* < 0.001 compared with *γ*-TPN group. In Figure 5(b), ^***^ *P* < 0.001 compared with vehicle, ^a^ *P* < 0.001 compared with the nicotine group, ^b^ *P* < 0.01 compared with *γ*-TPN group; + presents treatment; − presents missing treatment (one-way analysis of variance, Bonferroni's test).

As shown in Figure 4(a), naloxone significantly inhibited the antinociceptive effect of *γ*-TPN, as well as glibenclamide (Figure 4(b)). Figures 5(a) and 5(b) demonstrate the involvement of the cholinergic system. In Figure 5(a), atropine significantly inhibited the antinociceptive effect of *γ*-TPN, as well as mecamylamine (Figure 5(b)). However, pretreatment with L-arginine did not alter this effect (data not shown).

### 3.5. Measurement of Motor Performance

#### 3.5.1. Open Field and Rota Rod Test

In order to evaluate any nonspecific muscle-relaxant or sedative effects of *γ*-TPN, mice were submitted to the open-field and rota-rod test. In the test of open field, the *γ*-TPN (12.5 and 25 mg/kg, p.o.) did not alter the frequency of animals movement or the length of time the animals stayed in the bar in rota-rod test for 1 min when compared with vehicle (data not shown).

## 4. Discussion

Several studies have reported monoterpenes with a wide sort of biological properties, such as anti-inflammatory, anxiolytic, anticonvulsant, and antinociceptive [30–33]. Interestingly, this study demonstrates for the first time that the monoterpene *γ*-TPN promotes antinociceptive activity at low-range doses in different models of chemical nociception after oral administration, as well as spinal, supraspinal, or intraplantar in the glutamate-induced nociception model in mice.

For accuracy of pharmacological tests, the identification of the molecular mass of investigated compounds is strongly important due to surely confirming their chemical identities and the purity of the analyzed samples. Therefore, mass spectrometry analysis was performed by direct infusion in ion trap ESI positive mode. A pseudomolecular ion with *m*/*z* 137.0 Da [M + H]^+^ then confirms the identity and purity of *γ*-TPN samples [34].

The *γ*-TPN did not show any sign of acute oral toxicity in rats at the dose of 2 g/kg. Therefore, the 50% lethal dose (LD_50_) could not be determined. Accordingly, a previous work reported for *γ*-TPN low oral toxicity in rats, with LD_50_ of 3.65 g/kg [16]. The monitoring of the body weight is an important indicator for assessing the toxicity of a substance [35]. In turn, no significant changes in body weights were observed during 14 days after acute oral administration of *γ*-TPN. Furthermore, no changes in the relative weight of internal organs, as well as in serum biochemical parameters (ALT, AST, creatinine, and serum urea) and hepatic oxidative damage, were observed after treatment with *γ*-TPN, compared with vehicle. ALT, specific to hepatocytes, and AST, found in liver, cardiac muscle, and kidney, both are well-known as markers of cell damage, especially hepatocyte necrosis [36, 37]. Moreover, serum urea and creatinine are the most commonly used clinical markers of renal injury in routine toxicity studies [38]. Therefore, these data allowed a safe choice of the used doses for acute experimental protocols.

The *γ*-TPN was orally effective in inhibiting both phases of the formalin-induced nociception, which indicates the likely involvement of different mediators. The formalin-induced nociception test is commonly employed as a model of acute tonic pain, characterized by the presence of a distinct biphasic nociceptive response. The first phase corresponds to neurogenic pain by direct activation of the transient receptor, potentially A1 cation channels located at the sensory C-fibers, thus reflecting a centrally mediated pain [39]. The second phase of nociceptive response, also known as inflammatory pain, is mediated by a combination of peripheral input from inflammatory mediators released from injured tissues, causing the sensitization of central nociceptive neurones [40]. Previous reports point out the substance P and bradykinin participate in the appearance of the first-phase responses, while histamine, serotonin, prostagladin, and bradykinin are involved in the second-phase responses [40, 41]. Moreover, it is well established that drugs that act primarily on the central nervous system inhibit both phases equally, while peripherally acting drugs inhibit the second phase [40, 42]. Thus, *γ*-TPN could be acting by inhibition of direct and indirect acting inflammatory mediators and possibly affecting also the neurotransmission pathways at the SNC level (such as substance P or CGRP). Other plant essential oil-derived substances, such as bisabolol [43] and carvacrol [44], also had similar antinociceptive effects.

The present study also revealed that *γ*-TPN was effective in capsaicin-induced nociception model. Capsaicin is an alkaloid extracted from red pepper capsicum, which stimulates nociceptive and thermal nerve endings causing intense pain. Capsaicin acts specifically in unmyelinated type C fibers and poorly in myelinated and thin A*δ* fibers, at the vanilloid receptor (TRPV-1) in the peripheral nervous system, by opening a nonselective cation channel, mainly Ca^2+^ and Na^+^, and causing depolarization and initiation of action potentials. Capsaicin determines the release of neuropeptides, especially tachykinis (substance P, neurokinin B), which operate on transmission of pain sensation in nociceptive pathways and inflammatory processes [45]. Accordingly, the effect of the *γ*-TPN on the neurogenic phase of the formalin-evoked response was supported by the data obtained in the capsaicin test. Monoterpenes, such as carvacrol and citronellal, promote antinociceptive activity in the capsaicin test [10, 46].

Another interesting finding of this study is that *γ*-TPN, when administered by oral, intrathecal, intracerebroventricular, or intraplantar routes, protects the mice from glutamate-induced nociception in a significant manner, promoting not only peripheral, but also central action in this model. Glutamate is a major excitatory neurotransmitter which transmits nociceptive signals by promoting the direct activation of receptors in nociceptive fibers. Once activated, these neurons release several inflammatory mediators and neuropeptides that are involved in nociceptive transmission in the central and peripheral nervous system [47, 48]. Glutamate exerts its effects through both ionotropic and metabotropic glutamate receptors [49, 50]. Studies with the monoterpene linalool showed antinociceptive activity by the same routes [33]. Considering the structural similarity of these two monoterpenes, other studies corroborate our results showing that the linalool can also acting on the glutamatergic neurotransmission, since the NMDA receptor antagonism can cause supraspinal analgesia mediated by central opioid receptors stimulation [7, 51].

Therefore, in order to elucidate the possible antinociceptive mechanisms of *γ*-TPN, animals were pretreated with several drugs that interfere with different systems and evaluated on glutamate-induced nociception model.

The mechanism of action for *γ*-TPN, at least in part, seems to be from a direct action on the opioid receptors, since pretreatment of animals with naloxone inhibited the antinociceptive activity. The *γ*-TPN antinociceptive effect was also antagonized by pretreatment with glibenclamide, suggesting the involving of the opioid system via K^+^_ATP_ channels in *γ*-TPN antinociception. The opioid system is an important inhibitory system in nociception, which acts through two main pathways, central and peripheral. In central pathways opioid agonists act on the periaqueductal gray matter, rostroventromedial bulb, and dorsal horn of the spinal cord, activating the pain control descending pathways, in part, by activating potassium channels and inhibit voltage-dependent calcium channels [49].

Our results clearly indicate the involvement of cholinergic receptors (muscarinic and nicotinic) in this process, since atropine (nonselective muscarinic antagonist) and mecamylamine (a *α*2*β*3 selective preferential nicotinic receptor antagonist) inhibited the antinociceptive effect of *γ*-TPN. A major site of action for cholinomimetics in analgesia is the spinal cord. Painful stimuli are known to increase acetylcholine in the spinal cord. The activation of muscarinic receptors in the spinal cord results in the increased release of inhibitory transmitters and decreased release of excitatory transmitters, and this activation, in part, mediates the antinociceptive effect [52]. In addition, the results of this study provide strong evidence supporting the involvement of nicotinic receptors in the *γ*-TPN antinociception, since mecamylamine at a dose similar to that known to prevent antinociception induced by the selective agonist of the *α*2*β*3 nicotinic receptor, consistently attenuated both nicotine- (nicotinic receptor nonselective agonist) and the *γ*-TPN-induced antinociception in the glutamate test [53, 54].

Finally, another worthy finding of the present study was the demonstration that *γ*-TPN was largely devoid of significant effects on the motor performance of mice in the open field and rota-rod tests, thereby eliminating a nonspecific muscle relaxation and sedative effects in *γ*-TPN-induced antinociception.

## 5. Conclusions

The results presented here provided, for the first time, convincing evidence that oral administration of the monoterpene *γ*-TPN exerted pronounced antinociception when assessed in chemically induced nociception models in mice. In addition, *γ*-TPN has antinociceptive effect when administered into central (intracerebroventricular and intrathecal) and peripheral pathways, and this action occurs possibly with the involvement of the opioid system via K^+^_ATP_ channels and cholinergic system. So, this monoterpene seems to be a promising molecule as a future analgesic drug, taking into consideration the combination of efficacy and safety.

## Figures and Tables

**Table 1:** Antinociceptive effect of the *γ*-terpinene in the formalin-induced nociceptive response in mice.

Treatment	Dosage (mg/kg)	Licking time (s)			
		0–5 min	Inhibition (%)	15–30 min	Inhibition (%)
Vehicle	—	76.35 ± 10.21	—	70.68 ± 8.18	—

*γ*-TPN	6.25	50.48 ± 2.58	33.89	84.79 ± 12.78	−19.96
	12.5	5.96 ± 3.46^***^	92.20	24.13 ± 5.60^**^	65.87
	25	13.74 ± 4.08^***^	82.01	21.18 ± 6.72^***^	70.04

Morphine	5	18.19 ± 3.10^***^	76.18	17.32 ± 5.46^***^	75.50

Mice were treated with *γ*-terpinene (*γ*-TPN) 60 min (p.o.) before formalin test. Data represent the mean ± SEM of 6–9 mice. Statistical analysis was determined by one-way ANOVA followed by Tukey's test. ^**^ *P* < 0.01; ^***^ *P* < 0.001 compared with vehicle.
